# Enhanced Microstructure and Wear Resistance of Ti–6Al–4V Alloy with Vanadium Carbide Coating via Directed Energy Deposition

**DOI:** 10.3390/ma17030733

**Published:** 2024-02-03

**Authors:** Ui Jun Ko, Ju Hyeong Jung, Jung Hyun Kang, Kyunsuk Choi, Jeoung Han Kim

**Affiliations:** 1 Department of Materials Science & Engineering, Hanbat National University, Daejeon 34158, Republic of Korea; gej96@naver.com (U.J.K.); dong5675@naver.com (J.H.J.); wjdgus8627@naver.com (J.H.K.); 2 Department of Industry University Convergence, Hanbat National University, Daejeon 34158, Republic of Korea

**Keywords:** additive manufacturing, directed energy deposition, titanium alloy, vanadium carbide, wear resistance

## Abstract

Ti–6Al–4V alloys are known for their suboptimal tribological properties and are often challenged by durability issues under severe wear conditions. This study was conducted to enhance the alloy’s wear resistance by forming a hardened surface layer. Utilizing directed energy deposition (DED) additive manufacturing with a diode laser, vanadium carbide particles were successfully integrated onto a Ti–6Al–4V substrate. This approach deviates from traditional surface enhancement techniques like surface hardening and cladding, as it employs DED additive manufacturing under parameters akin to those used in standard Ti–6Al–4V production. The formed vanadium carbide layer achieved a remarkable thickness of over 400 µm and a Vickers hardness surpassing 1500 HV. Pin-on-disk test results further corroborated the enhanced surface wear properties of the Ti–6Al–4V alloy following the additive-manufacturing process. These findings suggest that employing vanadium carbide additive manufacturing, under conditions similar to the conventional DED process with a diode laser, significantly improves the surface wear properties of Ti–6Al–4V in metal 3D-printing applications.

## 1. Introduction

Titanium is renowned for its lightweight and high-strength attributes, and it possesses a range of beneficial properties including exceptional corrosion resistance, biocompatibility, ductility, a high melting point, and strength at elevated temperatures. These characteristics render titanium and its alloys highly valuable in diverse fields such as aerospace, biomedical devices, and chemical-plant construction [1,2,3,4,5,6,7,8]. Ti–6Al–4V is a commercially prominent titanium alloy that finds widespread application in various industries [9,10,11,12]. Despite their many advantages, titanium and its alloys fall short in abrasion resistance, particularly under abrasive corrosion conditions, limiting their applicability in certain scenarios [13]. To address this shortfall, enhancing the wear resistance of titanium and its alloys is of paramount importance. Traditional methods for surface enhancement, such as surface hardening [14] and surface cladding [13,14], have been utilized to improve their surface wear and erosion characteristics. In line with this, recent efforts have focused on advancing the surface properties of 3D-printed titanium components [15,16].

Metal additive-manufacturing (AM) processes are particularly suited for producing specialized, low-volume products. Titanium alloys, often employed in aerospace and biomedical implants, are frequently used in metal AM [17,18,19,20,21]. Common AM processes for titanium alloys include directed energy deposition (DED) and powder bed fusion (PBF), especially for repair work in the aerospace industry. Components produced via these methods are typically used immediately post-processing. However, post-manufacturing, titanium alloys retain inherent wear characteristics that often necessitate further processing for enhancement. If the wear properties of titanium alloy components fabricated through AM processes could be improved intrinsically, it would obviate the need for additional post-treatment steps such as cladding. This improvement could lead to significant reductions in both time consumption and energy expenditure.

The recent surge in interest towards fabricating functionally graded materials (FGMs) [22,23,24], which feature a gradual change in the mixture ratio of two or more materials based on location, has opened up new avenues in materials engineering. Titanium, with its high-strength and lightweight characteristics, emerges as an ideal candidate for incorporation into FGMs [23,24]. Its selection as a material in AM processes like DED and PBF has broadened its application spectrum. DED, in particular, is extensively used for its ability to deposit metal materials in a single process step, forming dense, thin-walled, or large-volume metal parts through the pneumatic injection of powder into a laser-induced melt pool. This technique has sparked numerous studies focused on fabricating FGMs, including heterogeneous metal systems, via AM technology for components unfeasible with traditional methods like welding [17,22]. Additionally, the use of high-power energy sources such as lasers in these processes facilitates the creation of unique microstructures, enhancing mechanical strength due to rapid heating and cooling.

In DED and PBF, lasers are chosen based on the metal’s energy absorption rate [25,26,27]. For metals like iron and titanium, the energy absorption rates exceed 30% when high-output energy diode lasers are used, ensuring efficient performance [28]. However, ceramics like silicon carbide show low energy-absorption rates with diode lasers, posing challenges in achieving temperatures above their melting points. However, in the DED process of depositing vanadium carbide (VC) powder on Ti–6Al–4V, the melted VC particles evenly distribute over the metal substrate, leading to the formation of a high-hardness surface layer on the titanium alloy.

This study’s objective was to enhance the wear characteristics of Ti–6Al–4V by utilizing DED to deposit VC powder on its surface, thereby creating an FGM. The study replicated the laser power and speed conditions used in the AM of titanium alloy and conducted a detailed examination of the melting pool, hardness, and wear characteristics of the Ti–6Al–4V substrate. This approach marks a significant stride in improving the durability of titanium alloys, utilizing the capabilities of AM technologies to develop advanced materials with superior wear properties.

## 2. Materials and Methods

**Figure 1:**
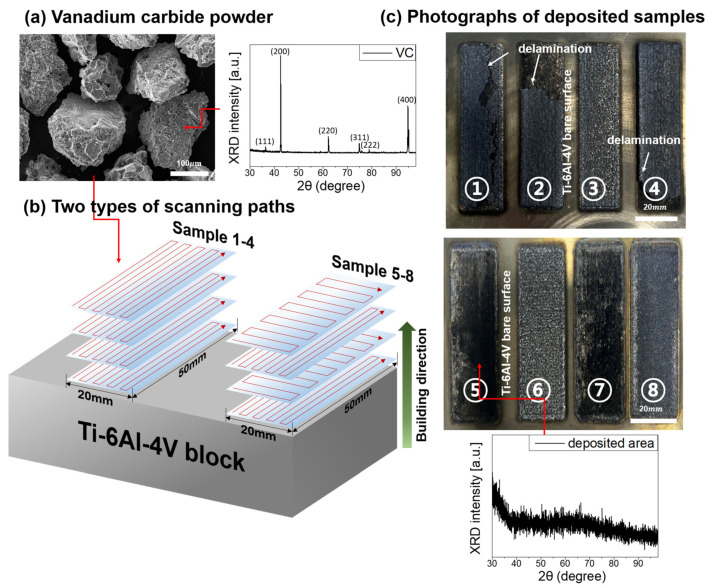
(**a**) Scanning electron microscopy (SEM) image of as-received VC powder. (**b**) Schematic depicting vanadium carbide (VC) powder scanning pass deposition on Ti–6Al–4V alloy. (**c**) Photos of deposited samples with insets of X-ray diffraction (XRD) patterns from as-received VC powder and deposited VC layer.

VC powder, sourced from Uninanotech Co., Ltd. (Yongin, Republic of Korea), underwent sieving to achieve a particle size distribution between 50 and 150 µm before the layering process, as depicted in Figure 1a. This size range was confirmed using scanning electron microscopy (SEM, Hitachi IT-500, Hitachi, Kyoto, Japan). Additionally, X-ray diffraction (XRD, SmartLab, Rigaku, Yamanashi, Japan) analysis was conducted, revealing that the VC powder was devoid of significant impurities.

In the DED process, two distinct building sequences were employed, as illustrated in Figure 1b. The substrate for the deposition consisted of a commercial Ti–6Al–4V bulk sample with a thickness of 50 mm, which was surface prepared through sanding with #600 grit emery paper. To prevent excessive heat-induced cracking, a raster pattern was initially applied for samples 1–4, considering the 20 mm short-side length of the deposition area. However, Cross-Sectional SEM imaging of these samples indicated the development of numerous voids post-deposition. In response, a zigzag pattern was adopted for samples 5–8 to address this issue. Notably, the deposition conditions were modified between the initial two layers and the subsequent layers for samples 5–8. Detailed deposition parameters for these two sample types are provided in Table 1.

The energy density (*E*) can be expressed as follows:

(1) E=(α×P)/(v×h×t)

*E* in the DED process for Ti–6Al–4V alloy substrates is a crucial factor, being directly proportional to the laser power *P* and the absorption coefficient *α*, while inversely proportional to scanning speed *v*, layer thickness t, and hatch spacing h. The absorption coefficient for the titanium substrate using a diode laser was fixed at 0.3. The values for layer thickness and hatch spacing were set at 0.025 cm and 0.6 cm, respectively. *E* was expressed in the units of J/cm³.

Sample cross-sections were analyzed using SEM and energy-dispersive X-ray spectroscopy (EDX). Wear characteristics of the deposited surfaces were evaluated with a Pin-on-Disk tester (Model No. RFW160, NEOPLUS, Daejeon, Republic of Korea) at 25 °C. In these tests, a silica ball was moved over a 20 mm range on the y-axis of the sample at 0.3 Hz for 2 h under a 5 N load. Prior to testing, surfaces were polished with #600 grit sandpaper for smoothness. The weight difference pre- and post-testing was measured using a sensitive electronic balance with 0.1 mg accuracy. For wear testing, four conditions were selected for samples 5–8 and the bare Ti–6Al–4V alloy.

Hardness was evaluated using a micro Vickers hardness tester (Duramin-40, Struers, Ottensoos, Germany) with a 100 gf load and 10 s dwell time. The Vickers hardness test was conducted thrice to obtain an average value, represented with error bars.

## 3. Results and Discussion

The study explored the impact of layering VC on Ti–6Al–4V alloy substrates under various conditions. As shown in Figure 1c, samples 1–4 experienced unexpected VC-layer delamination after the 3rd and 4th deposition attempts. This issue is attributed to the substantial difference in coefficients of thermal expansion (CTE) between VC and Ti–6Al–4V. The CTE of VC is around 5.0–6.0×10^−6^/°C, which is significantly lower than that of Ti–6Al–4V (8.6 × 10^−6^/°C) [29]. This discrepancy causes the alloy to undergo more pronounced thermal expansion during temperature fluctuations, potentially destabilizing the layered structure.

**Figure 2:**
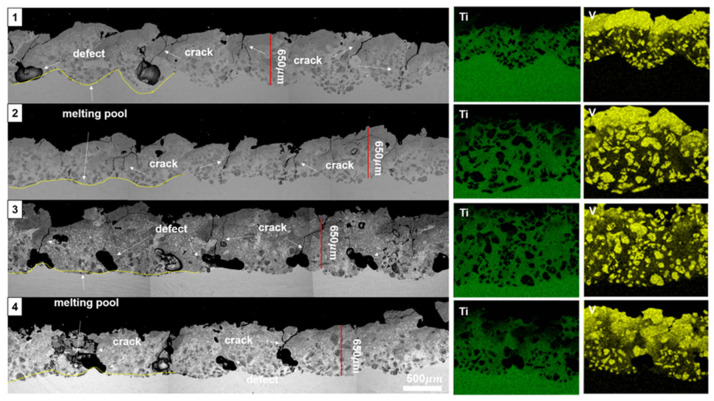
Scanning electron microscopy–backscattered electron detector (SEM–BED) and energy dispersive X-ray spectroscopy (EDS) mapping analysis, highlighting crack regions in samples 1–4.

To gain a more comprehensive understanding, samples 1–4 were subjected to detailed analyses using scanning electron microscopy–energy dispersive X-ray spectroscopy (SEM–EDS), as illustrated in Figure 2. Across all conditions, the melting pool formed on the alloy substrate exhibited an average depth of 500 µm. Notably, in samples 1, 2, and 4, where delamination was observed in Figure 1, it was found that the layer of pure VC deposition, distinct from the VC-Ti mixed layer, was considerably thick. This observation suggests that the presence of a pure VC layer contributes to delamination, primarily due to the residual stresses arising from the significant disparity in CTE between these layers and the titanium base. Given the small sample size, this might result in a localized concentration of high heat input, especially at points where the laser scanning paths overlap, leading to issues like inadequate fusion.

In an effort to address these challenges, a raster pattern was implemented in samples 1–4. However, this approach led to a new problem: an uneven distribution of VC particles and the consequent formation of defects. Further SEM–EDS analysis revealed that the laser deposition process created a melting pool of the alloy, into which VC particles were incorporated. This mixture then rapidly cooled to form the deposited layer. However, this process also led to pitch-to-pitch cracking, predominantly due to the differing CTEs between the ceramic VC and the metal alloy. Increased laser power exacerbated the delamination of the VC layer from the alloy substrate, leading to more pronounced cracks between pitches due to the accumulation of high-energy-driven VC particles. Along with this, the occurrence of deposition defects, such as holes, was observed to rise in proportion to the pitch-to-pitch cracks. However, the incidence of insufficient fusion between pitches decreased with higher laser power.

These findings underscore the critical need to strike a careful balance in controlling laser power, ensuring adequate pitch-to-pitch fusion, and managing VC particle distribution. This delicate interplay warrants further investigation to optimize the DED process for VC layering on Ti–6Al–4V substrates.

**Figure 3:**
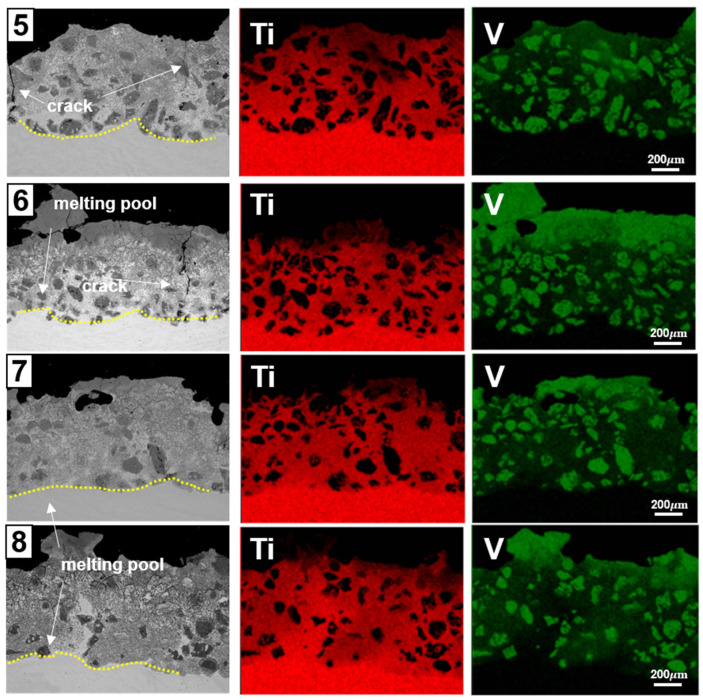
SEM–BED and EDS mapping analysis, showing crack regions of samples 5–8.

In the case of samples 5–8, a different and more favorable outcome was observed compared to samples 1–4. These samples underwent a similar preparation process, with an equal number of deposition layers. The SEM–EDS images of the cross-sections of samples 5–8, as shown in Figure 3, reveal a notable improvement. Unlike samples 1–4, samples 5–8 exhibited no signs of VC delamination. This improved outcome is attributed to the introduction of a zigzag laser-scanning pattern, specifically designed to minimize defects between pitches.

Contrasting with the issues faced in samples 1–4, samples 5–8 demonstrated enhanced interface quality and a reduced incidence of pitch-to-pitch defects. However, the complexity of the situation should not be overlooked. While samples 7 and 8 showed few surface cracks, samples 5 and 6 still exhibited cracking, particularly around areas of VC particle aggregation. This phenomenon was similar to what was observed in samples 1–4, despite the implementation of a zigzag scanning path and low-energy-density deposition conditions aimed at maintaining flatness between the Ti–6Al–4V substrate and the VC-deposited layer.

This situation presents a challenge: while the zigzag scanning approach seems to yield overall better results, it does not completely eliminate crack formation. Therefore, it offers a potential avenue for further research and process optimization but is not an outright solution to the problem. The persistence of cracks, especially in areas with concentrated VC particles, raises additional questions. The comparison of samples prepared using the zigzag scanning approach with those using a unidirectional scanning path adds another layer of complexity to understanding the interaction between the scanning pattern, VC particle distribution, and crack formation. This complexity underscores the need for continued research to optimize the additive-manufacturing process for VC deposition on Ti–6Al–4V substrates.

Despite the challenges, samples processed with the zigzag scanning technique showed a notable reduction in cracks and defects. XRD analysis of the layers deposited after the Ti-VC mixed layer revealed that the pure VC-deposition layers transitioned into an amorphous state, as indicated in the inset of Figure 1c. However, the exact mechanisms driving this transition to an amorphous state remain unclear. The atomic arrangement and bonding in VC, characterized by a mix of metallic and covalent bonds within its cubic crystal structure, might contribute to a more disordered arrangement, especially under rapid cooling conditions. Additionally, pure VC’s sensitivity to air at room temperature and its gradual re-oxidization [30,31], along with its potential reaction with oxygen in high-temperature environments created by diode lasers, leading to the formation of amorphous carbon [32], are factors worth considering.

**Figure 4:**
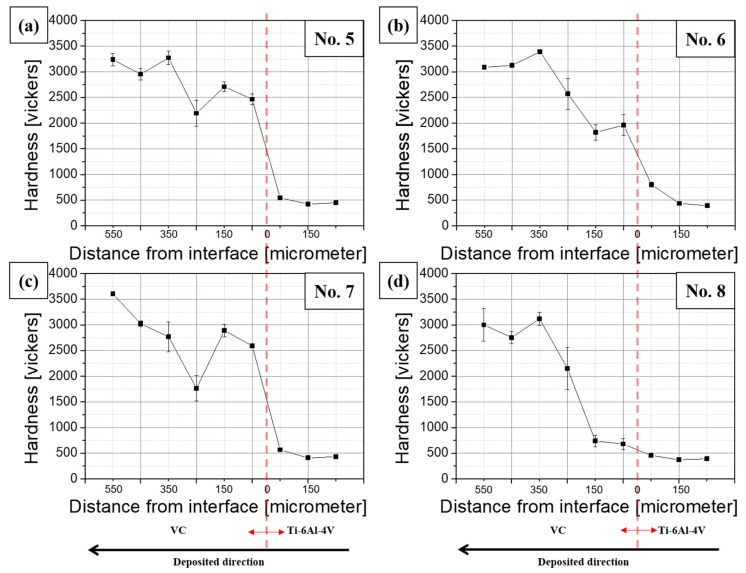
Vickers hardness results for samples (**a**) 5, (**b**) 6, (**c**) 7, and (**d**) 8. The red dotted lines indicate the interface between Ti–6Al–4V and VC.

A critical aspect of this research was examining the hardness of the deposited layers. As depicted in Figure 4, the Vickers hardness of the VC-deposited layers increased notably with the density and concentration of VC, exceeding an average of 3000 HV, with sample 7 displaying the highest hardness at 3604 HV. Vickers hardness measurements were performed on both the VC particles and the titanium alloy base, as seen in Figure 3. The hardness at the interface between the VC layer and the titanium alloy base was approximately 2000 HV, considerably higher than the 450 HV of the titanium alloy base alone. This enhancement in hardness could be attributed to the integration of VC particles into the molten Ti–6Al–4V substrate during the additive-manufacturing process and possibly to the formation of titanium carbide via the bonding of titanium metal with VC. However, the hardness enhancement may not be consistent across the entire deposited surface, owing to the variable distribution of VC particles caused by differing laser scanning paths. This potential for uneven hardness profiles could significantly impact the overall performance and durability of the alloy, highlighting the need for further investigation into optimizing the additive-manufacturing process for consistent material properties.

**Figure 5:**
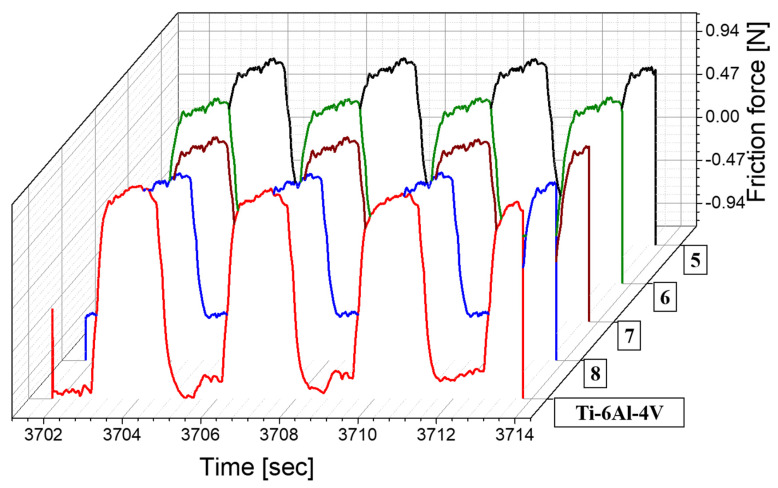
Pin-on-disk-test data for samples 5–8 (maroon, green, brown, and blue lines, respectively) and Ti–6Al–4V (red line).

The study further delved into analyzing the wear and friction characteristics of the alloy. To ensure accurate results, the surfaces of the samples were polished with emery paper, achieving a smooth finish suitable for testing. Notably, the friction force exhibited by samples 5–8 was approximately ±0.8 N, which was lower compared to the ±1.1 N observed in the Ti–6Al–4V alloy, as depicted in Figure 5. This difference in friction forces is crucial, particularly considering its impact on the wear resistance of the surface in practical applications.

Additional insights were obtained from pin-on-disk friction and wear tests. The friction coefficient for surfaces with VC deposition was found to be below 0.13, representing a significant improvement over the Ti–6Al–4V alloy, which demonstrated a friction coefficient exceeding 0.18, as indicated in Table 2. Notably, the wear results for VC after the abrasion test showed exceptionally low weight loss, indicative of minimal wear. In the pin-on-disk test, while the base material Ti–6Al–4V alloy exhibited a weight loss of 1.0 mg, the VC deposition either had a weight loss of 0.1 mg or no significant change in weight. Despite some uncertainty due to the limitations of weight sensitivity, this observation suggests enhanced wear characteristics.

The slight variations observed in the VC samples are attributed to the influence of pre-treatment grinding. The layered surfaces were polished using #600 grit sandpaper to ensure flatness prior to the wear test. Significantly, EDX mapping results (Figure 3) revealed that samples 5, 7, and 8 contained areas with a Ti metal matrix on the surface, while sample 6 exhibited a surface with a pure VC layer. It was observed that the VC layer in sample 6, lacking the Ti metal matrix, developed cracks due to thermal expansion post-deposition. Furthermore, the weight reduction of sample 6 after the wear test is believed to result from the delamination of the pure VC layer, underscoring the complex interplay between the material composition, surface treatment, and wear performance.

**Figure 6:**
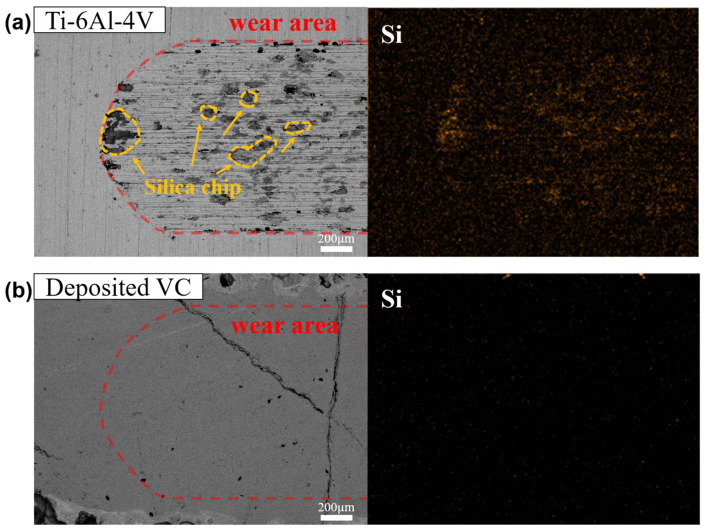
SEM–BED and EDX mapping analysis of wear surfaces; (**a**) bare Ti–6Al–4V, and (**b**) Ti–6Al–4V with VC deposition.

Samples 5, 7, and 8, showing no weight change after the pin-on-disk test, demonstrate that the VC-Ti mixed layer resulting from VC deposition markedly improves surface friction and wear characteristics. This enhancement has the potential to extend the lifespan and boost the performance of the material in a range of applications. However, these findings also highlight the intricate nature of the deposition process and the complex material properties that result from it. To gather deeper insights into the wear mechanism, an SEM analysis was conducted on the surfaces of the wear track following the pin-on-disk test, as presented in Figure 6. Analyzing the wear specimen is vital for understanding the wear behavior, and notable differences were observed between the bare Ti–6Al–4V alloy and the VC-deposited surface. Figure 6a depicts the worn surface of the Ti–6Al–4V alloy post-wear test, while Figure 6b shows the worn surface of sample 7, which exhibited the highest surface hardness. The Ti–6Al–4V alloy was subject to wear by a silica ball during the test, leading to adhesive wear due to differences in bond strength at the contact area between silica and the alloy. SEM–EDS mapping analysis also revealed tiny Si-containing chips on the worn Ti–6Al–4V surface, indicative of abrasive wear. In stark contrast, the VC-deposited surface showed no significant morphological changes due to the silica ball, underscoring its resistance to wear.

The notable improvements in hardness and friction characteristics resulting from VC deposition suggest that fine tuning the deposition process can offer substantial benefits. However, the consistency of these results is not guaranteed, as they are likely influenced by variables such as VC concentration, laser scanning path, and heat input. A more comprehensive understanding of these factors and their interplay is crucial to optimize the deposition process and its outcomes effectively. Additional research is needed to confirm these results and elucidate the underlying mechanisms responsible for these improvements, paving the way for the development of more robust and durable materials through advanced additive-manufacturing techniques.

## 4. Conclusions

The study successfully deposited VC onto a Ti–6Al–4V alloy surface using DED. This process involved an argon shield gas environment, the implementation of two distinct scanning paths to minimize defects, and a VC powder with particle sizes ranging from 50 to 150 µm. The primary findings of this study can be summarized as follows:

1. VC particles were embedded within a molten Ti–6Al–4V matrix without complete melting by optimizing the laser and power settings, given VC’s high melting point. However, subsequent layers of pure VC were prone to delamination due to thermal expansion differences.
2. Denser regions were formed through interconnections between VC particles. However, these areas were more susceptible to interlayer cracks. A less-continuous arrangement of VC particles might help alleviate this problem.
3. The zigzag scanning path in DED proved more effective than the raster pattern in minimizing defects.
4. Sample 7, subjected to specific deposition conditions, demonstrated the highest surface hardness. The friction coefficient across the samples showed minimal variation.
5. The deposition of VC over Ti–6Al–4V via DED using a diode laser extends the alloy’s application scope without necessitating alterations to existing conditions. However, a comprehensive understanding of the outcomes and the complex interplay among various process parameters requires further investigation.

This study opens avenues for optimizing Ti–6Al–4V alloy properties for enhanced performance in various applications and underscores the need for continued research to fully harness this potential.

## Figures and Tables

**Table 1:** Laser deposition parameters by layer sequence.

Sample No.	Laser Power [W]	Laser Speed [mm/min]	Energy Density× 10^3^ [J/cm^3^]	Layers
1	800	1000	9.6	5
2	800	500	19.2	5
3	500	1000	6.0	5
4	500	500	12.0	5
5	500	1000	6.0	1–2
	500	800	7.5	3–5
6	500	1000	6.0	1–2
	500	500	12.0	3–5
7	500	1000	6.0	1–2
	800	500	19.2	3–5
8	500	1000	6.0	1–2
	800	1000	9.6	3–5

**Table 2:** Overview of friction coefficients for VC-deposited samples.

Sample Name	Weight Loss [mg]	Average Friction Coefficient
Sample 5	0.0	0.1264
Sample 6	0.1	0.1275
Sample 7	0.0	0.1201
Sample 8	0.0	0.1244
Ti–6Al–4V (bare)	1.0	0.1864

## Data Availability

Data are contained within the article.
